# Carnitine Palmitoyltransferase 1B 531K Allele Carriers Sustain a Higher Respiratory Quotient after Aerobic Exercise, but β3-Adrenoceptor 64R Allele Does Not Affect Lipolysis: A Human Model

**DOI:** 10.1371/journal.pone.0096791

**Published:** 2014-06-06

**Authors:** Eduardo Gómez-Gómez, Martín Efrén Ríos-Martínez, Elena Margarita Castro-Rodríguez, Mario Del-Toro-Equíhua, Mario Ramírez-Flores, Ivan Delgado-Enciso, Ana Lilia Pérez-Huitimea, Luz Margarita Baltazar-Rodríguez, Gilberto Velasco-Pineda, Jesús Muñiz-Murguía

**Affiliations:** 1 Centro Universitario de Investigaciones Biomédicas, Universidad de Colima, Colima, Colima, México; 2 Facultad de Medicina, Universidad de Colima, Colima, Colima, México; 3 Educación Física y Deporte. Facultad de Ciencias de la Educación, Universidad de Colima, Colima, Colima, México; University of Milan, Italy

## Abstract

Carnitine palmitoyltransferase IB (CPT1B) and adrenoceptor beta-3 (ADRB3) are critical regulators of fat metabolism. CPT1B transports free acyl groups into mitochondria for oxidation, and ADRB3 triggers lipolysis in adipocytes, and their respective polymorphisms *E531K* and *W64R* have been identified as indicators of obesity in population studies. It is therefore important to understand the effects of these mutations on ADRB3 and CPT1B function in adipose and skeletal muscle tissue, respectively. This study aimed to analyze the rate of lipolysis of plasma indicators (glycerol, free fatty acids, and beta hydroxybutyrate) and fat oxidation (through the non-protein respiratory quotient). These parameters were measured in 37 participants during 30 min of aerobic exercise at approximately 62% of maximal oxygen uptake, followed by 30 min of recovery. During recovery, mean respiratory quotient values were higher in K allele carriers than in non-carriers, indicating low post-exercise fatty acid oxidation rates. No significant differences in lipolysis or lipid oxidation were observed between R and W allele carriers of *ADRB3* at any time during the aerobic load. The substitution of glutamic acid at position 531 by lysine in the CPT1B protein decreases the mitochondrial beta-oxidation pathway, which increases the non-protein respiratory quotient value during recovery from exercise. This may contribute to weight gain or reduced weight-loss following exercise.

## Introduction

A sedentary lifestyle has contributed to a substantial increase in the prevalence of overweight and obesity [1], [2], [3]. Although aerobic exercise can reduce excess body fat [4], [5], genetic factors may determine the susceptibility of an individual to gain weight [6], [7] and alter the beneficial effects of physical activity [8]. Aerobic exercise increases adipose tissue lipolysis, which releases free fatty acids (FFA) into the bloodstream, FFA uptake by active skeletal muscle, and FFA oxidation in the mitochondrial matrix [9].

Fatty acid oxidation during physical activity is strongly dependent on both the rate of lipolysis in the adipocytes [9], [10] and membrane translocation of long chain fatty acids (LCFA) into the mitochondrial matrix of the muscle cell [9], [11]. Corpeleijn et al. demonstrated that intramyocellular lipid oxidation depends on extracellular FFA availability as well as mitochondrial function [12]. It has been documented, that FFA concentrations increase during the recovery period after exercise at 65% of maximal oxygen uptake (VO_2_max), which releases more FFA for oxidation in skeletal muscle. This is supported by the decline in non-protein respiratory quotient (RQnp) value during recovery [13].

The beta-3 adrenoceptor (*ADRB3*) gene is expressed in visceral and subcutaneous adipose tissue and encodes the catecholamine receptor that activates lipolysis [14]. A polymorphic variant of *ADRB3* is located on chromosome 8p12 [15] with the substitution of tryptophan (W) by arginine (R) at position 64 (*W64R*). The R allele has been associated with excessive abdominal fat accumulation, higher body mass index (BMI), lower high density lipoprotein (HDL) levels, and insulin resistance [14], [16], [17]. However, in two population studies conducted on the effect of physical activity, the results were not different between W and R allele carriers [18], [19].

Carnitine palmitoyltransferase 1 (*CPT1*; chromosome 22q13.33), expressed in skeletal muscle, heart, and the testis [20], regulates long-chain acyl group entry into the mitochondria for incorporation into the beta-oxidation pathway [9], [20]. Robitaille et al. found a significant association between the heterozygous *E531K* allelic variant genotype, which replaces glutamic acid (E) for lysine (K) at position 531 of muscle-type CPT1 (*CPT1B*), and indicators of obesity when the proportion of dietary fat was ≥34.4%, indicating a gene–diet interaction [11]. Another study conducted with people of African descent in Trinidad and Tobago found that K allele homozygote carriers had a lower percentage of intermuscular fat, but a higher percentage of subcutaneous fat when compared with heterozygous and homozygous E allele carriers [21]. Moreover, an association between the K allele and metabolic syndrome traits was found in an adult German population [22].

We hypothesized that under aerobic effort, R allele carriers of *ADRB3* would present lower plasma concentrations of glycerol (Gly) and FFA, whereas K allele carriers of *CPT1B* would present higher values of RQnp. Indices of lipolysis and fat oxidation can also help determine whether the R and K alleles interact to reduce the fat oxidative capacity. This study aimed to determine the influence of these genetic variations R allele on lipolysis and that of the K allele on fatty acid oxidation by comparing carriers and non-carriers during and after aerobic load. Results indicate that the *E531K* affect fatty acid oxidation regardless of *W64R*.

## Methods

The Ethics and Biosafety Committee of Colima University reviewed and approved the study protocol in accordance with the Declaration of Helsinki. All participants provided written informed consent.

### Participants

In total, 45 participants were included from a group of 285 individuals who had recently participated in a study of the association between markers of obesity and genetic polymorphisms. Participants had normal hemoglobin, blood glucose, blood lipid, and lipoprotein levels, and fulfilled the additional inclusion criteria of BMI values and proportions of dietary macronutrients (carbohydrates, fats, and proteins) within the average range [mean±1 standard deviation (SD)] established in the association study (unpublished data). The proportions of dietary macronutrients were obtained by 24 h recall. Data were analyzed using Diet Analyst software (Parsons Technology Inc NUTRIDATA Software Corporation). We also calculated the quantities of saturated, monounsaturated, and polyunsaturated fatty acids (SFA, MUFA, and PUFA, respectively) in the diet adjusted to the body weight.

Subcutaneous whole body fat was determined from sum of 9 skin folds (∑9P); this included triceps, subscapular, biceps, iliac crest, supraspinal, abdominal, front thigh, and medial calf in accordance with the guidelines and equipment of the International Society for the Advancement of Kinanthropometry (ISAK), [23] as well as forearm skinfold measured on the distal lateral line at maximum forearm girth [24]. Subcutaneous trunk fat was determined from the sum of 4 trunk skinfolds (Σ4S); this included subscapular, iliac crest, supraspinal, and abdominal [23], and abdominal adiposity was determined by waist circumference (WC) measured in accordance with ISAK guidelines and equipment [23]. None of the participants had undertaken an exercise program during the past year, and none had received drug or hormonal treatments during the past 6 months.

### Evaluation of Cardiovascular Function

Evaluation was performed by a cardiologist. All participants were apparently healthy; however, clinical examination revealed respiratory or cardiovascular anomalies in some participants, and these underwent further review with echocardiography and electrocardiography resulting in the exclusion of 3 participants who required treatment.

### Cardiopulmonary Fitness

The Tecumseh step test was used to assess fitness on a 20.3-cm high step. According to its guidelines, participants performed 24 steps/min for 3 min and maintained a constant stepping rate with the aid of a metronome and assistant. Heart rate (HR) monitors (Polar-mod A-5 902805, Kempele, Finland) [25] recorded participant HRs prior to exercise, and in the recovery phase at 1 and 3 min post-exercise. The HR value at 1 min was classified in accordance with established categories [26].

### Genomic DNA

The sodium dodecyl sulfate (SDS)–proteinase K method was used to extract genomic DNA from white blood cells, using a total of 7 mL of venous blood collected in two 5 mL tubes with EDTA (Ethylenediaminetetraacetic acid, 0.25 M). Briefly, cells were washed thrice in ammonium chloride (131 mM) plus ammonium bicarbonate (0.9 mM) with intervening centrifugation (15 min, 5000 revolutions per minute (rpm), 4°C), digested in proteinase buffer (50 mM Tris, pH 7.5; 100 mM EDTA, pH 8.0; 400 mM sodium chloride (NaCl); 0.5% SDS; and 50 µL of 20 mg/mL proteinase K), and agitated for 12 h at 37°C. To separate the DNA from protein, 6 M NaCl was added under centrifugation (15 min, 10000 rpm, 4°C). The supernatant (3−4 mL) was mixed with 15 mL absolute ethanol to condense the DNA. The DNA was transferred to 1.5-mL Eppendorf tubes containing 1 mL of 70% ethanol and then centrifuged for 10 min at 7500 rpm. Ethanol was removed by evaporation using a vacuum system, and the DNA pellet was resuspended in TE solution (10 mM Tris, pH 7.5; 1 mM EDTA) at 4°C for 12 h. DNA level was quantified by absorbance spectrophotometry at 260 nm. Aliquots (100 µL) at 100 ng/µL DNA were stored at 4°C until used for genotyping.

### Genotyping of Polymorphisms

The *W64R* and *E531K* were genotyped by polymerase chain reaction–restriction fragment length polymorphism (PCR–RFLP) analysis. The reference single nucleotide polymorphisms (refSNPs) for *W64R* and *E531K* are rs4994 and rs470117, respectively [27], [28]. Primers were designed using Primer 4.0 (http://frodo.wi.mit.edu) as follows: F-AGCCAGGTGATTTGGGAGAC and R-GTCACACACAGCACGTCCAC for amplification of a 429-base pair (bp) fragment including *W64R* and F-GCACTTCCCTCTTACCCACA, and R-CTTCTTGATGAGGCCTTTGC for amplification of a 311-bp fragment including *E531K*, which were synthesized by Invitrogen Custom DNA Oligos (Life Technologies).

The hybridization temperature was 60°C for *W64R* and 63°C for *E531K*. Amplification was performed in 35 cycles, each comprising 30 s denaturation at 94°C, 30 s annealing at 60°C or 63°C, and 30 s elongation at 72°C. The PCR product for *W64R* was digested for 16 h in 5 U Pf1MI enzyme (New England Bio Labs. R0509S. Lot: 0280905), which recognizes the tryptophan base of the W allele and cuts the product into two fragments of 252 and 177 bp. After digestion, the full 429-bp fragment is therefore from the R allele. The PCR product for *E531K* was digested for 16 h in 5 U *Taq*I (New England Bio Labs R0149S. Lot: 0540804), which recognizes the glutamic acid base of the E allele and cuts the product into two fragments of 217 and 94 bp. Thus, the full 311-bp fragment is from the K allele. Digestion products were separated by 6% polyacrylamide gel electrophoresis and stained with 0.2% silver nitrate.

### Blood Chemistry Analyses

Venous blood samples were taken from the antecubital vein 12 h after fasting [29], approximately 5–6 weeks before the aerobic load. The parameters measured were (mg/dL): total cholesterol (T-Chol), triacylglycerol (TAG), high density lipoprotein cholesterol (HDL-C), and low density lipoproteins cholesterol (LDL-C) using standardized colorimetric methods (Spinreact SAU Ctra Santa Coloma, 7E-17176 Sant Esteve de Bas. GI Spain, spectrophotometer: Jenway model 6405 UV/Vis). In addition, hemoglobin (Hb) level was measured in 0.04 mL of whole blood diluted in Drabkin reagent (Hycel de México SA de CV), the hematocrit (Ht) percentage was determined by capillary tube centrifugation (10000 rpm for 5 min), and the glucose (Glc) concentration was measured from a 0.6 µL capillary blood sample using a portable glucometer (Bayer, CONTOUR TS, No Coding, Ascensia MICROLET, Japan).

### Maximal Oxygen Uptake Value

VO_2_max was determined using a standardized maximum exercise effort protocol on a treadmill, according to the recommendations of the American College of Sport Medicine [30]. A Quark b2. 8.0 bV oximeter (Cosmed, Rome, Italy) was used to measure oxygen (O_2_) consumption and carbon dioxide (CO_2_) exhalation during testing. Each participant performed a simulation of the test for 4–7 days prior to the stress test, so that they were aware of the effort required. Participants were advised to avoid consuming alcohol and caffeine as well as avoid smoking and vigorous physical activity for at least 48 h prior to testing. The stress test was performed 3–4 h after food intake.

### The Aerobic Load

**Figure 1:**
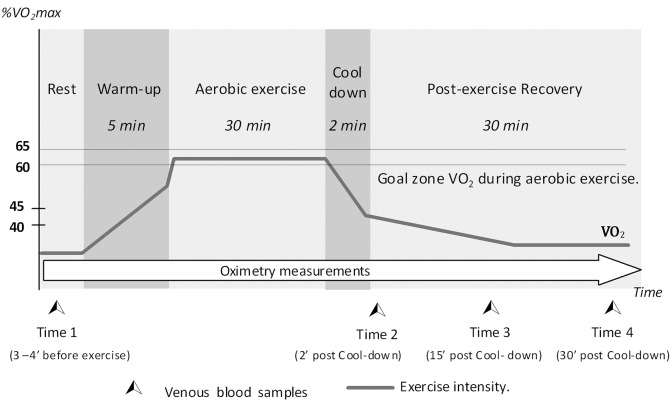
Aerobic load structure (5 exercise periods and 4 blood sample Times) and exercise intensity programme.

This consisted of a 30-min exercise on a treadmill, followed by recovery in the supine position for 30 min. To achieve greater precision in load development, the test was structured with five periods as follows: (a) Rest, 6–10 min sitting at rest; (b) Warm-up, 5-min running on the treadmill to gradually reach 40% of VO_2_max; (c) Aerobic exercise, 30 min at 60% of VO_2_max; (d) Cool-down, 2 min of gradually reducing the running speed to zero; and (e) Post-exercise recovery, 30 min at rest in the supine position. (Figure 1).

The CO_2_ production (VCO_2_) and O_2_ uptake (VO_2_) were measured throughout the stress test. RQnp, absolute oxygen consumption (VO_2_ in mL/min), relative oxygen consumption (VO_2_ in mL/kg/min), and relative CO_2_ production (VCO_2_ in mL/Kg/min) were calculated during all 5 aerobic load periods.

Venous blood samples were collected from the cubital vein at 4 times: sample 1, at 3−4 min before the warm-up; sample 2, at 2 min post-exercise recovery; sample 3, at 15 min post-exercise recovery; and sample 4: at 30 min post-exercise recovery (Figure 1). Blood samples were used to determine levels of the following: epinephrine (Epn), norepinephrine (Nep), insulin, glucagon, cortisol (Cor), growth hormone (GH), Gly, FFAs, beta-hydroxybutyrate (beta-HB), Glc, lactate (Lac), albumin (ALB), and Ht. Plasma hormone level measurements were performed by the UNIDAD DE PATOLOGIA CLINICA (México Av. 2341. Guadalajara, México), a laboratory certified by The College of American Pathologist (AV-ID: 1200227. LAP: 5832901). Glc and Ht (%) levels were measured as described in the blood chemistry section. Lac levels were measured using a portable lactometer (Accutrend Plus System, Roche Diagnostics, San Cugat del Vallès, Barcelona Spain). Standard colorimetric methods were used to determine beta-HB, Gly, and FFA levels (Randox Laboratories Ltd, Ardmore, Diamond Road, Crumlin, Co. Antrim, UK, BT29 4QY) and ALB levels (SAU Spinreact Ctra Santa Coloma, 7E-17176 Sant Esteve de Bas, GI Spain).

The aerobic challenge was performed 10−20 days after maximum exercise testing to determine VO_2_max. Women participants performed the aerobic load test within the first 10 days of their menstrual cycle.

### Blood Hormone Levels

Samples were taken once before the aerobic load challenge and thrice during recovery (2, 15, and 30 min in post-exercise recovery period) (Figure 1). At each sampling point, 16 mL of venous blood was obtained and used as follows: (a) 8 mL in 2 tubes (4 mL each) with sodium heparin to determine Epn and Nep by chemiluminescence assay, (b) 4 mL in cold EDTA for determining plasma glucagon levels by radioimmunoassay, (c) 4 mL in a dry tube for determining serum insulin and Cor levels by electrochemiluminescence immunoassay and GH by chemiluminescence assay.

### Statistical Analyses

We used SPSS version 15 (IBM SPSS Software, USA) for all analyses. The effects of *E531K* and *W64R* on the oximetry values at the end of each of the 16-measurement stages and blood hormone levels were tested both separately and together (interaction). The normality and variance homogeneity of each distribution were determined by the Kolmogorov–Smirnov and Levene tests, respectively. The values are presented in tables as mean ± SD. The mean values at each stage and time point were compared between K allele carriers vs. K allele non-carriers (K+ and K−, respectively), R allele carriers vs. R allele non-carriers (R+ and R−, respectively), and, carriers of both (K+/R+), vs. carriers of K only (K+/R−) or R only (R+/K−). For normal and homogeneous distributions, one-way analysis of variance and post-hoc Tanhame tests for pairwise comparisons were used. Otherwise, the Kruskal–Wallis test was used to compare 3 distributions, and the Mann–Whitney U-test for pairwise comparisons. The statistical significance level was set at *p*<0.05.

## Results

### Participant Characteristics

Of the 45 individuals selected, only 37 (18 men, 19 women) successfully completed the experiment; 3 were excluded because of heart problems and 5 were lost to follow up (they relocated before the study was completed). All parents and grandparents were born in Mexico, with 35 originating from Western Mexico and two from South–Central Mexico. (Table S1 in File S1). The clinical characteristics were as follows: age range, 22.9±3.1 years; height, 162.7±7.8 cm; weight, 63.2±10.4 kg; BMI, 23.8±3.1 kg/m^2^; systolic blood pressure, 115.49±13.4 mmHg; and diastolic blood pressure, 71.6±6.9 mmHg.

### Data Sorting

All functional measurements were grouped and compared according to the genetic variant studied, as follows: (a) R+ (20; 10 males, 10 females) versus (vs.) R− (17; 8 males, 9 females) to assess the effect of *W64R*; (b) K+ (27; 13 males, 14 females) vs. K− (10; 5 males, 5 females) to assess the effect of *E531K*; and (c) K+/R+ (12; 6 males, 6 females) vs. K+/R− (15; 7 males, 8 females) vs. K−/R+ (8; 4 males, 4 females) to test for an interaction between alleles. Only two participants (1 male and 1 female) were K−/R−; therefore, this genotype was not included in the statistical comparison (Table S2 in File S1).

### Anthropometric, Dietary, and Blood Characteristics

There were no significant differences in age or adiposity indices (BMI, WC, Σ9S, Σ4S, and WHR) between genotypes within the 3 comparison groups (R+ vs. R−; K+ vs. K−; K+/R+ vs. K+/R− vs. K−/R+*; p*>0.05) (Table 1). Furthermore, the mean dietary protein, fat, carbohydrate, dietary SFA/kg, and MUFA/kg intakes did not significantly differ between groups. The amount of PUFA/kg was marginally higher for K− than for K+ (*p*<0.070) (Table 2). Measured levels of Glc, Ht, Hb, TAG, T-Chol, HDL-C, and LDL-C were not significantly different between genotypes within the comparison groups (*p*>0.05), indicating that basal metabolic status was similar (Table 3). The mean HR values at 1 min after exercise in the Tecumseh step test did not significantly differ between any groups (Table S3 in File S1), establishing similar levels of cardiopulmonary fitness between the groups.

**Table 1:** Mean (SD) values of age, BMI and indicators of subcutaneous fat in each of the genetic classifications.

Genetic Classification	Groups	Age (years)	BMI (kg/m^2^)	WC (cm)	∑9S (mm)	∑4S (mm)
CPT1B alleles	K+	23.1 (3.4)	24.2 (3.2)	77.3 (8.1)	150.7 (50.5)	60.2 (22.0)
	K−	22.5 (2.1)	22.7 (2.7)	75.7 (8.3)	143.9 (50.5)	55.5 (21.1)
		***^A^*** *p = 0.663*	***^A^*** *p = 0.182*	***^A^*** *p = 0.618*	***^A^*** *p = 0.720*	***^A^*** *p = 0.562*
ADRB3 alleles	R+	22.4 (1.9)	23.6 (3.7)	76.8 (8.5)	148.2 (53.1)	57.5 (22.7)
	R−	23.5 (4.1)	24.0 (2.4)	76.8 (7.8)	149.6 (47.4)	60.6 (20.7)
		***^A^*** *p = 0.266*	***^A^*** *p = 0.659*	***^A^*** *p = 0.996*	***^A^*** *p = 0.935*	***^A^*** *p = 0.668*
Interaction ADRB3–CPT1B	K+/R+	22.3 (1.9)	24.8 (4.1)	79.0 (8.5)	159.6 (55.8)	61.1 (23.5)
	K+/R−	23.6 (4.3)	23.7 (2.2)	75.8 (7.8)	143.5 (46.4)	59.5 (21.5)
	R+/K−	22.5 (2.1)	21.7 (1.8)	73.6 (7.8)	131.1 (46.9)	52.2 (21.9)
		***^K–W^*** *p = 0.973*	***^K–W^*** *p = 0.051*	***^K–W^*** *p = 0.192*	***^K–W^*** *p = 0.402*	***^K–W^*** *p = 0.669*

***SD*** *:* Standard deviation. ***A*** *:* One-way analysis of variance (ANOVA), significance *p*<0.05. ***K–W***: Kruskal–Wallis test, significance p<0.05.

**BMI**: Body mass index. **WC**: Waist circumference. **∑9S**: Sum of triceps, subscapular, biceps, iliac crest, supraspinal, abdominal, anterior thigh, medial calf, and forearm skin folds. **∑4S**: Sum of subscapular, iliac crest, supraspinal, and abdominal skin folds. (Skinfold of trunk). **K+**: K allele carriers. **K−**: K allele non-carriers. **R+**: R allele carriers. **R−**: R allele non-carriers. **K+/R+**: K and R allele carriers. **K+/R−**: K allele only carriers. **R+/K−**: R allele only carriers.

**Table 2:** Mean macronutrient proportions and dietary fatty acid amounts adjusted to body weight in each of the genetic classifications.

Genetic Classification	Groups	Protein (%)	C–H (%)	Fat (%)	SFA/kg	MUFA/kg	PUFA/kg
CPT1B alleles	K+	17.3 (3.2)	53.1 (7.8)	30.7 (5.5)	0.39 (.14)	0.36 (.12)	0.17 (.06)
	K−	15.8 (3.1)	56.7 (3.6)	29.1 (2.6)	0.40 (.11)	0.40 (.14)	0.21 (.08)
		***^A^*** *p = 0.212*	***^U^*** *p = 0.203*	***^U^*** *p = 0.353*	***^A^*** *p = 0.801*	***^A^*** *p = 0.411*	***^A^*** *p = 0.070*
ADRB3 alleles	R+	16.6 (2.6)	54.5 (4.9)	30.2 (3.3)	0.39 (.13)	0.37 (.13)	0.18 (.08)
	R−	17.2 (3.8)	53.7 (9.2)	30.4 (6.4)	0.39 (.14)	0.37 (.12)	0.18 (.05)
		***^A^*** *p = 0.555*	***^U^*** *p = 0.964*	***^U^*** *p = 0.940*	***^A^*** *p = 0.956*	***^A^*** *p = 0.882*	***^U^*** *p = 0.879*
Interaction ADRB3–CPT1B	K+/R+	17.0 (2.3)	53.6 (5.7)	30.4 (3.8)	0.37 (.14)	0.33 (.12)	0.15 (.07)
	K+/R−	17.5 (3.8)	52.7 (9.4)	30.9 (6.6)	0.40 (.14)	0.38 (.12)	0.18 (.05)
	R+/K−	16.0 (3.1)	55.8 (3.3)	29.8 (2.4)	0.42 (.11)	0.43 (.13)	0.22 (.09)
		***^K–W^*** *p = 0.719*	***^K–W^*** *p = 0.421*	***^K–S^*** *p = 0.567*	***^K–S^*** *p = 0.789*	***^K–S^*** *p = 0.178*	***^K–S^*** *p = 0.284*

***SD*** *:* Standard deviation. ***A***: One-way analysis of variance (ANOVA): significance, p<0.05. ***U***: Mann–Whitney test: significance, p<0.05. ***K–W***: Kruskal–Wallis H test: significance, p<0.05.

**C–H**: Carbohydrates. **SFA/kg**: Saturated fatty acids per kg of body weight. **MUFA/kg**: Mono unsaturated fatty acids per kg of body weight. **PUFA/kg**: Poly unsaturated fatty acids per kg of body weight. **K+**: K allele carriers. **K−**: K allele non-carriers. **R+**: R allele carriers. **R−**: R allele non-carriers. **K+/R+**: K and R allele carriers. **K+/R−**: K allele only carriers. **R+/K−**: R allele only carriers.

**Table 3:** Mean fasting blood glucose, hematocrit, hemoglobin, and lipid levels in each of the genetic classifications.

Genetic Classification	Groups	Glc (mg/dL)	Ht (%)	Hb (mg/dL)	TAG (mg/dL)	T-Chol (mg/dL)	HDL-C (mg/dL)	LDL-C (mg/dL)
CPT1B alleles	K+	87.3 (9.6)	42.5 (3.8)	13.1 (1.6)	99.9 (25.5)	150.7 (24.6)	39.6 (10.8)	131.4 (49.7)
	K−	82.2 (7.2)	42.9 (4.6)	14.1 (2.1)	94.5 (45.8)	141.5 (23.9)	39.3 (7.4)	132.9 (49.6)
		***^A^*** *p = 0.136*	***^A^*** *p = 0.797*	***^A^*** *p = 0.171*	***^A^*** *p = 0.648*	***^A^*** *p = 0.316*	***^A^*** *p = 0.940*	***^A^*** *p = 0.932*
ADRB3 alleles	R+	83.4 (7.9)	43.4 (3.1)	13.1 (1.5)	92.7 (33.0)	147.0 (21.8)	38.3 (9.2)	130.3 (48.5)
	R−	88.9 (10.0)	41.7 (4.7)	13.7 (2.0)	105.3 (29.5)	149.7 (27.9)	41.0 (10.7)	133.5 (51.0)
		***^A^*** *p = 0.074*	***^A^*** *p = 0.214*	***^A^*** *p = 0.308*	***^A^*** *p = 0.235*	***^A^*** *p = 0.743*	***^A^*** *p = 0.408*	***^A^*** *p = 0.849*
Interaction ADRB3–CPT1B	K+/R+	83.6 (9.3)	43.6 (3.0)	12.8 (1.6)	94.8 (23.1)	151.5 (22.2)	38.7 (10.6)	134.8 (49.0)
	K+/R−	90.3 (9.1)	41.6 (4.1)	13.4 (1.6)	104.1 (27.4)	150.1 (27.2)	40.4 (11.2)	128.6 (51.8)
	R+/K−	83.3 (6.1)	43 (3.3)	13.5 (1.4)	89.6 (45.8)	140.2 (20.6)	37.7 (7.3)	123.7 (50.4)
		***^A^*** *p = 0.091*	***^K–W^*** *p = 0.722*	***^A^*** *p = 0.119*	***^A^*** *p = 0.645*	***^A^*** *p = 0.777*	***^A^*** *p = 0.736*	***^A^*** *p = 0.693*

***SD*** *:* Standard deviation. ***A***: One-way analysis of variance (ANOVA), significance p<0.05. ***K–W***: Kruskal–Wallis test, significance p<0.05.

**Glc**: Glucose. **Ht**: Hematocrit. **Hb**: Hemoglobin. **T-Chol**: Total cholesterol. **HDL-C**: High density lipoprotein-cholesterol. **LDL-C**: Low density lipoprotein-cholesterol. **K+**: K allele carriers. **K−**: K allele non-carriers. **R+**: R allele carriers. **R−**: R allele non-carriers. **K+/R+**: K and R allele carriers. **K+/R−**: K allele only carriers. **R+/K−**: R allele only carriers.

### Oximetry Indicators

**Figure 2:**
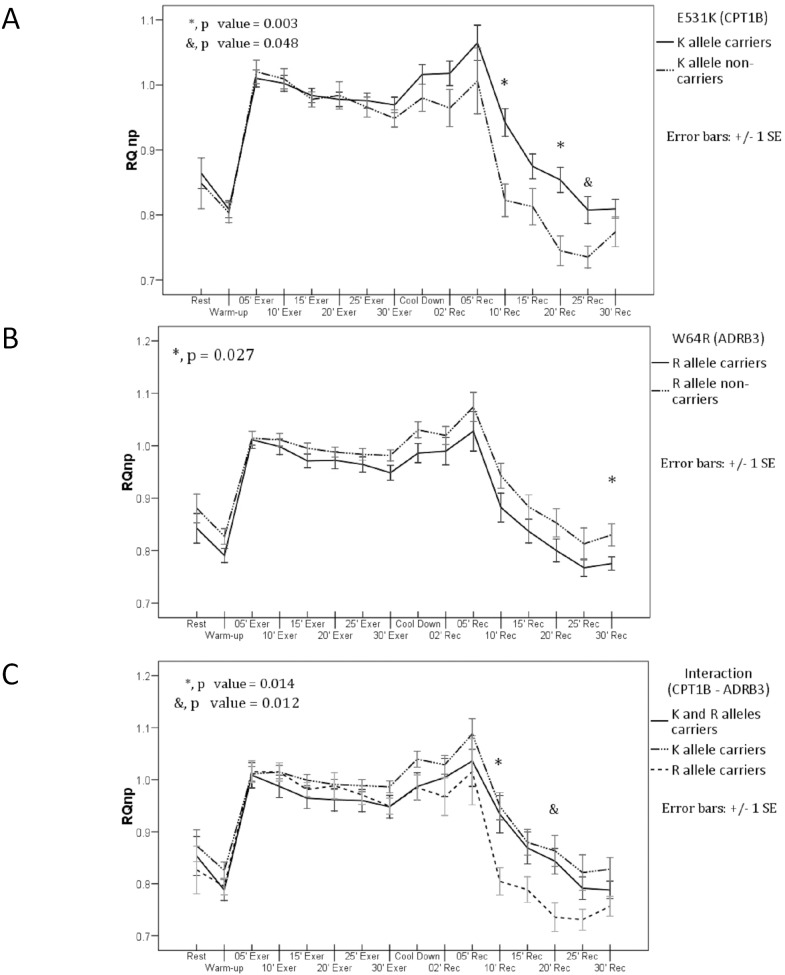
Non-protein respiratory quotient (RQnp) values during aerobic load. (**A**) R allele carriers (12 K and 8 E carriers) versus R allele non-carriers (15 K and 2 E carriers). (**B**) K allele carriers (12 R and 15 W allele carriers) versus K allele non-carriers (2 R and 8 W carriers) The asterisk and ampersand indicate a significant difference. (**C**) Interaction (*CPT1B–ADRB3*). The asterisk and ampersand indicate the significant difference between the R allele carrier group, with 8 carriers of the E allele, and the other groups.

The VO_2_max percentage (%VO_2_max) was taken as the main indicator of exercise intensity. Participants performed the warm-up phase to 35.5%±5.3% of VO_2_max for 5 min, the aerobic exercise phase at 62.3%±1.1% of VO_2_max for 30 min, and the cool-down phase at 42.2%±4.6% VO_2_max for 2 min. A significant difference was detected between R+ and R− groups (63.1% vs. 61.3%; *p* = 0.027) at 10 min into the aerobic exercise phase (Table 4). Despite similarities in baseline metabolic values, fitness levels, exercise intensity, and post-exercise hormone levels (below), there were significant differences in RQnp values between genotypes, specifically between K+ and K− groups (Figure 2A) with either R+ or R− (Figure 2C). The RQnp values were not significantly different between genotypes within the comparison groups for the rest, warm-up, aerobic exercise, and cool-down periods, but the RQnp value in the K+ group remained significantly higher at 10 (*p* = 0.003), 20 (*p* = 0.003), and 25 min (*p* = 0.048) after exercise when compared with the K− group. Moreover, the RQnp value was significantly higher in the R− than in the R+ participants at 30 min after exercise (*p* = 0.027) (Figure 2B). The R+/K− group had an RQnp value that was significantly lower than that of the K+/R+ and K+/R− groups at 10 min after exercise (*p* = 0.022), and significantly lower than that of the K+/R− group at 20 min after exercise (*p* = 0.028) (Figure 2C) (Table 5). Mean HR were no significantly different between genotypes throughout the exercise and recovery periods (*p*>0.05) (data not shown).

**Table 4:** Mean percentage of maximal oxygen uptake during and after the aerobic load for each of the genetic classifications.

Classification		Stages of the aerobic load															
		Rest	Warm-up	5′exercise	10′exercise	15′exercise	20′exercise	25′exercise	30′exercise	Cool down	2′ post exercise	5′ post exercise	10′ post exercise	15′ post exercise	20′ post exercise	25′ post exercise	30′ post exercise
**%VO_2_max**	**K+**	11.9	35.2	59.8	62.1	62.8	62.5	62.8	62.2	41.9	25.7	12.9	11.0	10.5	10.4	10.4	10.2
		(3.5)	(4.8)	(4.1)	(2.2)	(2.1)	(2.8)	(2.5)	(2.1)	(3.4)	(2.8)	(1.9)	(1.4)	(1.4)	(1.7)	(1.6)	(1.7)
	**K–**	13.3	36.1	60.8	63.9	62.4	63.1	64.0	63.8	43.2	26.1	11.9	11.9	11.6	11.4	11.2	11.2
		(2.6)	(6.6)	(8.7)	(5.5)	(7.1)	(8.2)	(7.5)	(6.7)	(7.1)	(5.4)	(4.9)	(2.9)	(2.8)	(3.2)	(3.1)	(2.7)
	*** p***	*0.274*	*0.637*	*0.658*	***^U^*** *0.608*	*0.817*	*0.744*	***^U^*** *0.538*	***^U^*** *0.707*	*0.457*	***^U^*** *0.682*	*0.380*	*0.211*	***^U^*** *0.180*	***^U^*** *0.502*	***^U^*** *0.458*	*0.195*
	**R+**	12.4	36.3	60.8	63.1*	63.1	63.0	63.9	63.0	42.5	25.8	12.9	11.7	11.3	11.3	11.0	11.0
		(2.7)	(5.7)	(6.3)	(4.0)	(5.2)	(6.2)	(5.6)	(4.9)	(5.6)	(4.1)	(3.6)	(2.1)	(2.0)	(2.4)	(2.3)	(2.3)
	**R−**	12.1	34.5	59.2	61.3*	62.2	62.4	62.2	62.2	41.9	25.8	12.4	10.9	10.2	10.0	10.2	9.9
		(4.0)	(4.7)	(4.7)	(1.8)	(1.9)	(2.4)	(2.0)	(2.3)	(3.1)	(3.0)	(2.0)	(1.7)	(1.7)	(1.8)	(1.8)	(1.4)
	*** p***	*0.750*	*0.319*	*0.399*	***0.027***	*0.509*	*0.688*	*0.254*	*0.537*	*0.673*	*0.989*	*0.675*	*0.213*	*0.090*	*0.081*	*0.246*	*0.116*
	**K+/R+**	12.1	36.5	61.1	63.2	63.4	62.8	63.3	62.2	42.0	25.5	13.5	11.2	10.8	11.0	10.5	10.6
		(2.6)	(4.6)	(2.5)	(2.0)	(2.2)	(3.3)	(2.9)	(1.9)	(3.7)	(2.9)	(1.9)	(1.6)	(1.2)	(1.8)	(1.7)	(2.0)
	**K+/R−**	11.8	34.2	58.8	61.2	62.2	62.3	62.3	62.2	41.8	25.8	12.5	10.9	10.2	9.9	10.3	9.9
		(4.1)	(4.9)	(4.8)	(1.9)	(2.0)	(2.5)	(2.1)	(2.4)	(3.2)	(2.8)	(1.9)	(1.6)	(1.5)	(1.5)	(1.6)	(1.3)
	**R+/K−**	13.0	35.9	60.3	64.4	62.5	63.3	64.7	64.1	43.3	26.2	11.9	12.3	12.0	11.7	11.7	11.5
		(2.9)	(7.3)	(9.8)	(6.1)	(8.0)	(9.3)	(8.3)	(7.5)	(7.9)	(5.7)	(5.3)	(2.9)	(2.6)	(3.1)	(3.0)	(2.8)
	*** p***	*0.705*	*0.679*	*0.671*	*0.142*	*0.880*	*0.976*	*0.608*	*0.692*	*0.899*	*0.984*	*0.671*	*0.374*	*0.200*	*0.330*	*0.349*	*0.343*

SD: Standard deviation. One-way analysis of variance (ANOVA), significance p<0.05. ***U***: Mann–Whitney test, significance p<0.05. Asterisk (*) indicates the values that significantly differ in the same stage.

**K+**: K allele carriers. **K−**: K allele non-carriers. **R+**: R allele carriers. **R−**: R allele non-carriers. **K+/R+**: K and R allele carriers. **K+/R−**: K allele only carriers. **R+/K−**: R allele only carriers.

**Table 5:** Mean non-protein respiratory quotients during and after the aerobic load for each of the genetic classifications.

Classification		Stages of the aerobic load															
		Rest	Warm-up	5′exercise	10′exercise	15′exercise	20′exercise	25′exercise	30′exercise	Cooldown	2′ postexercise	5′ postexercise	10′ postexercise	15′ postexercise	20′ postexercise	25′ postexercise	30′ postexercise
**RQnp**	**K+**	0.864	0.809	1.010	1.002	0.984	0.978	0.976	0.969	1.016	1.018	1.065	0.942*	0.875	0.854*	0.808*	0.810
		(0.12)	(0.07)	(0.07)	(0.06)	(0.06)	(0.06)	(0.06)	(0.06)	(0.08)	(0.10)	(0.14)	(0.11)	(0.10)	(0.10)	(0.11)	(0.07)
	**K−**	0.849	0.804	1.020	1.009	0.978	0.984	0.966	0.949	0.980	0.965	1.006	0.823*	0.813	0.745*	0.735*	0.774
		(0.12)	(0.05)	(0.06)	(0.05)	(0.04)	(0.07)	(0.05)	(0.04)	(0.07)	(0.09)	(0.16)	(0.08)	(0.09)	(0.07)	(0.05)	(0.07)
	*** p***	*0.736*	*0.818*	*0.677*	*0.758*	*0.766*	*0.790*	*0.639*	*0.335*	*0.205*	*0.143*	*0.286*	***0.003***	*0.090*	***0.003***	***0.048***	*0.210*
	**R+**	0.843	0.791	1.011	0.998	0.971	0.972	0.964	0.949	0.986	0.990	1.028	0.882	0.837	0.800	0.767	0.775*
		(0.13)	(0.06)	(0.07)	(0.07)	(0.06)	(0.07)	(0.06)	(0.06)	(0.08)	(0.12)	(0.17)	(0.12)	(0.10)	(0.10)	(0.07)	(0.06)
	**R−**	0.881	0.827	1.015	1.011	0.995	0.988	0.984	0.982	1.030	1.020	1.074	0.943	0.883	0.853	0.813	0.830*
		(0.11)	(0.06)	(0.06)	(0.05)	(0.04)	(0.04)	(0.05)	(0.04)	(0.06)	(0.07)	(0.11)	(0.10)	(0.09)	(0.11)	(0.12)	(0.08)
	*** p***	*0.346*	*0.078*	*0.888*	*0.504*	*0.166*	*0.436*	*0.309*	*0.079*	*0.076*	***^U^*** *0.270*	*0.343*	*0.111*	*0.160*	*0.131*	*0.173*	***0.027***
	**K+/R+**	0.853	0.788	1.009	0.987	0.965	0.962	0.960	0.948	0.987	1.004	1.036	0.934	0.869	0.843	0.792	0.788
		(0.13)	(0.07)	(0.08)	(0.08)	(0.07)	(0.07)	(0.07)	(0.08)	(0.09)	(0.13)	(0.17)	(0.12)	(0.11)	(0.08)	(0.08)	(0.06)
	**K+/R−**	0.873	0.826	1.011	1.015	0.999	0.991	0.989	0.986	1.040	1.029	1.088	0.949	0.880	0.863*	0.822	0.828
		(0.12)	(0.06)	(0.06)	(0.05)	(0.04)	(0.04)	(0.04)	(0.04)	(0.06)	(0.07)	(0.11)	(0.10)	(0.09)	(0.11)	(0.13)	(0.08)
	**R+/K−**	0.827	0.795	1.016	1.015	0.981	0.989	0.971	0.949	0.985	0.968	1.015	0.805*	0.789	0.736*	0.731	0.757
		(0.13)	(0.05)	(0.06)	(0.05)	(0.04)	(0.07)	(0.05)	(0.04)	(0.07)	(0.10)	(0.18)	(0.07)	(0.07)	(0.08)	(0.06)	(0.05)
	*** p***	*0.664*	*0.369*	*0.944*	*0.613*	*0.373*	*0.598*	*0.542*	*0.276*	*0.173*	*0.470*	*0.556*	***0.022***	*0.143*	***0.028***	*0.212*	*0.125*

SD: Standard deviation. One-way analysis of variance (ANOVA), significance p<0.05. ***U***: Mann–Whitney test, significance p<0.05. Asterisk (*) indicates the values that significantly differ in the same stage.

**K+**: K allele carriers. **K−**: K allele non-carriers. **R+**: R allele carriers. **R−**: R allele non-carriers. **K+/R+**: K and R allele carriers. **K+/R−**: K allele only carriers. **R+/K−**: R allele only carriers.

### Blood Indicators of Aerobic Load

**Figure 3:**
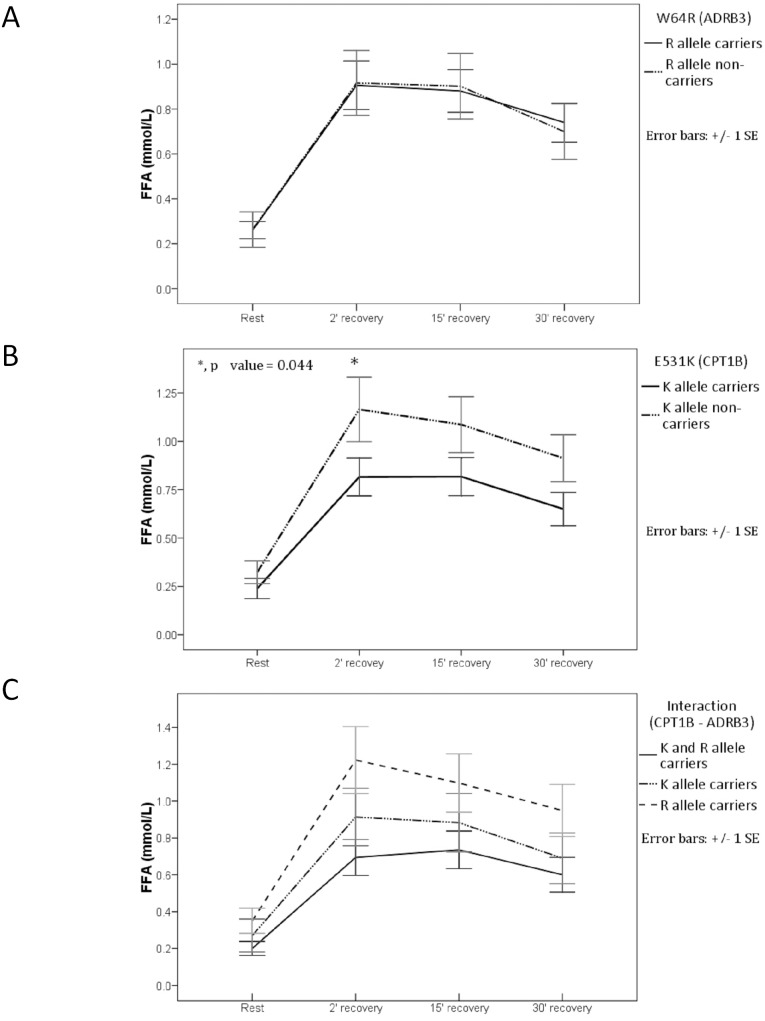
Free fatty acid (FFA) levels before and after the aerobic load. (**A**) R allele carriers (12 K and 8 E carriers) versus R allele non-carriers (15 K and 2 E carriers). (**B**) K allele carriers (12 R and 15 W allele carriers) versus K allele non-carriers (2 R and 8 W carriers). The asterisk indicates significant difference. (**C**) Interaction (*CPT1B–ADRB3*), carriers of both K and R alleles versus only K and only R allele carriers. Venous blood was sampled at rest and at 2, 15, and 30 min into the recovery period.

**Figure 4:**
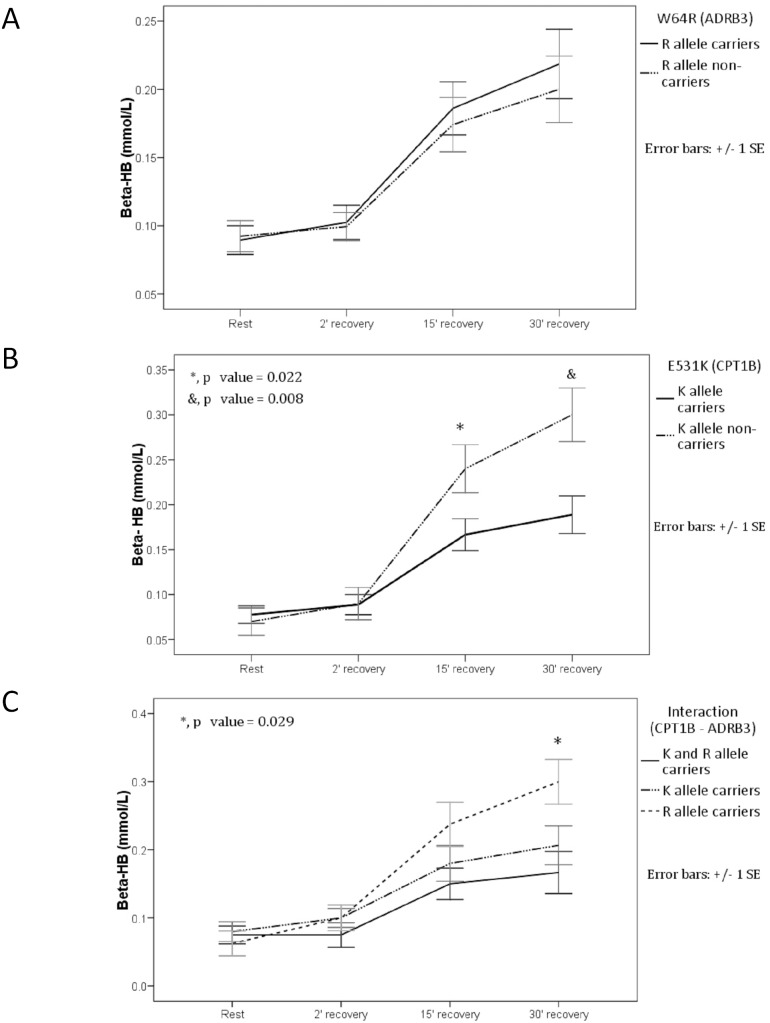
beta-Hydroxybutyrate (beta-HB) levels before and following the aerobic load. (**A**) R allele carriers (12 K and 8 E carriers) versus R allele non-carriers (15 K and 2 E carriers). (**B**) K allele carriers (12 R and 15 W allele carriers) versus K allele non-carriers (2 R and 8 W carriers). The asterisk indicates significant difference. (**C**) Interaction (*CPT1B–ADRB3*), carriers of both K and R alleles versus only K and only R allele carriers. The asterisk indicates a significant difference between the R allele group with 8 carriers of the E allele and the other groups. Venous blood was sampled at rest and at 2, 15, and 30 min into the recovery period.

Lac levels were not significantly different between genotypes within the 3 comparison groups prior to testing and at 2, 15, and 30 min after exercise (*p*>0.05). Lac levels exceeded 2 mM only at 2 min after exercise, and lower values were measured at 15 and 30 min. Glc levels decreased slightly during the post-exercise recovery phase, but not significant differences were observed between the genotypes. Similarly, ALB levels and Ht were not significantly different between genotypes at any stage (Table S4 in File S1). The K− group had significantly higher FFA levels than those in the K+ group at 2 min after exercise (*p* = 0.044) (Figure 3B; Table 6), whereas there were no significant differences in Gly levels between genotypes at any of the 4 measurement points. Beta-HB levels tended to increase during the post-exercise recovery phase (Table 6); however, no significant differences were found between the R+ and R− participants (Figure 4A), and levels were significantly higher at 15 min (*p* = 0.022) and 30 min (*p* = 0.008) after exercise in the K− than in the K+ participants (Figure 4B). Furthermore, a significantly higher beta-HB level was detected at 30 min after exercise in the K−/R+ group compared with either the K+/R+ or K+/R− groups (*p* = 0.029) (Figure 4C).

**Table 6:** Mean free fatty acid and beta-hydroxy butyrate at rest and at 2, 15, and 30 min post-exercise for each of the genetic classifications.

Genetic Classification		Rest	2′ post	15′ post	30′ post
			exercise	exercise	exercise
FFA (mM)	**K+**	0.24 (0.21)	0.82 (0.51)*	0.82 (0.51)	0.65 (0.45)
	**K−**	0.32 (0.19)	1.16 (0.53)*	1.09 (0.46)	96.2 (0.38)
		*^U^p = 0.067*	***^U^p = 0.044***	*^U^p = 0.139*	*^U^p = 0.062*
	**R+**	0.26 (0.17)	0.91 (0.48)	0.88 (0.42)	0.74 (0.39)
	**R−**	0.26 (0.32)	0.92 (0.59)	0.90 (0.60)	0.70 (0.51)
		*^A^p = 0.972*	*^A^p = 0.954*	*^A^p = 0.901*	*^A^p = 0.785*
	**K+/R+**	0.20 (0.13)	0.69 (0.34)	0.74 (0.35)	0.60 (0.33)
	**K+/R−**	0.27 (0.35)	0.91 (0.61)	0.88 (0.61)	0.69 (0.53)
	**R+/K−**	0.35 (0.19)	1.22 (0.51)	1.10 (0.45)	0.95 (0.40)
		*^A^p = 0.627*	*^A^p = 0.189*	*^A^p = 0.464*	*^A^p = 0.414*
beta-HB (mM)	**K+**	0.079 (0.05)	0.089 (0.06)	0.167 (0.09)*	0.189 (0.11)*
	**K−**	0.070 (0.05)	0.090 (0.06)	0.240 (0.08)*	0.300 (0.09)*
		*^U^p = 0.699*	*^U^p = 0.950*	***^U^p = 0.022***	***^U^p = 0.008***
	**R+**	0.070 (0.05)	0.085 (0.06)	0.185 (0.09)	0.220 (0.12)
	**R−**	0.082 (0.05)	0.094 (0.06)	0.188 (0.10)	0.218 (0.11)
		*^U^p = 0.491*	*^U^p = 0.618*	*^U^p = 0.961*	*^U^p = 0.987*
	**K+/R+**	0.075 (0.05)	0.075 (0.06)	0.150 (0.08)	0.167 (0.11)
	**K+/R−**	0.080 (0.06)	0.100 (0.05)	0.180 (0.10)	0.207 (0.11)
	**R+/K−**	0.062 (0.05)	0.100 (0.05)	0.240 (0.09)	0.300 (0.09)**
		***^K^*** *p = 0.756*	***^K^*** *p = 0.444*	***^K^*** *p = 0.098*	***^K^p = 0.029***

***SD*** *:* Standard deviation. ***A*** *:* One-way analysis of variance (ANOVA). ***U***: Mann–Whitney test. ***K***: Kruskal–Wallis test. Significance p<0.05. Asterisk (*) indicates the values that significantly differ in the same stage. Double asterisk (**) indicates that the value differs significantly from the other values in the same stage.

**FFA**: Free fatty acids. **beta-HB**: Beta hydroxybutyrate. **K+**: K allele carriers. **K−**: K allele non-carriers. **R+**: R allele carriers. **R−**: R allele non-carriers.

### Plasma Hormone Levels during Aerobic Load

Insulin, Cor, and GH levels did not differ significantly between genotypes within the comparison groups at any time. Glucagon levels remained below 50 pg/mL at all 4 measurement times. Epn and Nep levels reached peak levels at 2 min after exercise (84.81 and 676.14 pg/mL, respectively) and declined thereafter. The only significant difference in Nep level was detected at 30 min after exercise between R+ and R− participants (230.6 pg/mL vs. 301.7 pg/mL; *p* = 0.028) (Table S5 in File S1). In general, the hormonal responses were as expected and were generally similar among the genotypes.

## Discussion

The increasing rate of fatty acid oxidation induced by aerobic exercise is modulated by multiple factors. In this study, we therefore sought to reduce the influence of other polymorphic genes involved in the lipolysis and fatty acid oxidation pathway by including only Mexicans from only 2 generations, and primarily from the same region of Western Mexico.

Carriers of the K allele exhibited a higher mean RQnp during the post-exercise recovery phase, indicating reduced lipid beta-oxidation compared with non-carriers. In light of previous studies demonstrating an association between the K allele and obesity indices [11], this study suggests that the K allele may predispose to decreased FFA oxidation when the energy demand from lipids is increased during the recovery from effort. This can be a factor contributing to the accumulation of fat in adipose tissue given similar dietary and activity conditions to non-carriers.

The difference in RQnp between K allele carriers and non-carriers cannot be explained by differences in diet, general physical condition, or demographic valuables (sex ratio or age). Expression of the human *CPT1B* gene in transgenic mice increased 2-3-fold during both fasting and high-fat diets [31], whereas PUFA modulate the expression of genes involved in lipid metabolism, such as the peroxisome proliferator activated receptor [32]. Thus, dietary factors can influence the expression of *CPT1B* and other genes involved in fat mobilization. However, the comparison groups reported similar dietary macronutrient compositions (including SFA, MUFA, and PUFA). In addition, the level of cardiopulmonary fitness did not differ between genotypes (Table S3 in File S1); thus, obviating the differential effects of adaptation to physical activity [33], [34]. Moreover, subcutaneous fat, Glc, Hb, lipid, lipoprotein, and Ht levels did not differ between the comparison groups, indicating that metabolic conditions were similar at baseline. During the development of aerobic load, there were no differences in %VO_2_max among the genotypes. Moreover, blood Lac levels did not reach 4 mM at any point after exercise, which is indicative of aerobic metabolism (Table S4 in File S1). These results confirmed that exercise load was predominantly aerobic and of similar intensity among all participants.

Hormone levels involved in the modulation of lipolysis and fat oxidation did not differ significantly among the genotypes after exercise. Furthermore, ALB, Hb, and Ht levels were not significantly different. The apparent differences in lipid metabolism between K allele carriers and non-carriers was therefore unlikely to be due to differences in hormonal patterns, basal FFA, or oxygen transport and FFA transport capacity to active muscle tissue.

No significant differences were observed in plasma FFA and Gly levels between R allele carriers and non-carriers. Moreover, the similarity in catecholamine and other hormone levels before and after exercise indicates that the R allele does not affect the activation of lipolysis; these genotypes probably had similar FFA metabolism rates. There was a trend toward high values among R+ in RQnp, but only reached significant difference at 30 minute post-exercise recovery period compared to non-carriers (possibly related to the 12 K allele carriers) and a downward trend among non-carriers (possibly related to the 15 E allele carriers). In addition, it has been observed that LCFA oxidation in the mitochondria increases with FFA availability [9], [35], which was similar in both groups. In addition, beta-HB levels did not differ between R allele carriers and non-carriers during recovery. These results indicate that the availability of FFA, mobilized from visceral adipose tissue was similar to its consumption by skeletal muscle tissue.

Most participants in this study were heterozygous for the R allele, which already been reported in other studies [10], [14], [16], [18]. Thus, the gene dose may have contributed to the similarity in lipolysis indicators between R allele carriers and non-carriers. In addition, these indices were measured for only 30 min after exercise; delayed responses may therefore exist with measureable differences. Another possibility is that the effect of the *W64R* variant of *ADRB3* could be masked by the effect of other lipolysis agents, including cytokines that are activated during muscle contraction [36]. Nonetheless, several studies and meta-analyses have shown that although the R allele is associated with increased BMI and insulin resistance, its contribution to the phenotype is weak [14], [17], [38]. Our results indicate that the *W64R* allelic variant of *ADRB3* does not affect the activation of lipolysis.

The RQnp values during the post-exercise recovery phase were higher in K allele carriers (Figure 1-B) and independent of the R allele (Figure 1-C). This is consistent with the hypothesis that the K allele affects the translocation capacity of CPT1B, and that the effect is manifested under increased energy demands from fat that occur at the end of a prolonged workout. K allele carriers showed higher RQnp values during recovery, suggesting contributions to energy generation from fat equivalents of 19.3% at 10 min, 49.3% at 15 min, and 63.1% at 25 min compared with 59.7%, 88.0%, and 91.6% at the same post-exercise times in non-carriers [4]. Thus, K allele carriers demonstrate significantly lower fat metabolism than non-carriers. It is unlikely that the higher RQnp values among K allele carriers resulted from a reduced availability of circulating FFA because there were no differences in plasma FFA concentrations between groups after exercise, in addition the Gly values were similar, and there were no differences in hormone levels or exercise intensity. However, this is an opportunity for future research. Napal et al. [39] determined that the in vitro substitution of glutamic acid at position 590 of CPT1A increased sensitivity to malonyl coenzyme A. They concluded that the glutamic acid residue may impart structural stability to the protein, and thus affect the functional properties of the whole CPT family. In this study, we found that the substitution of glutamic acid at position 531 by lysine in the CPT1B protein had a significant effect on RQnp increase.

Notably, the sample size of this study was too small to include sufficient numbers with the rare K−/R− genotype; therefore, we could only assess the effects of allelic interaction among carriers of both alleles (K+/R+) and those carrying only one of the alleles (K+/R− and K−/R+). Consequently, the K−/R+ group also functioned as the non-carrier group for the K allele. During the recovery period, FFA levels were higher in the K−/R+ group than in the other two groups, although the difference was not statistically significant. At 10 and 20 min after exercise, this group also showed significantly lower RQnp values (Figure 2-C), and at 30 min after exercise showed significantly higher beta-HB levels (Figure 4-C). These observations indicate that the *E531K* had significant effects that were independent of *W64R*.

In this experimental design, the observation of the influence of the *E531K* SNP of *CPT1B* was facilitated because the CPT1 protein represents the only route of entry of LCFA into the beta-oxidation pathway. In contrast, ADRB3 lipolysis is modulated by the receptor as well as by several hormonal and metabolic factors. In this study, we ensured only that there were no significant differences in plasma levels of catecholamines, insulin, glucagon, Cor and growth hormone, lipids, diet (particularly PUFA content), exercise intensity, body adiposity, and cardiorespiratory fitness. Therefore, further studies are needed to strengthen and clarify the functional impact of the gene mutations involved in lipid metabolism, together with other factors such as the levels of interleukin-6 produced by muscle during contraction that can induce lipolysis within adipose tissue that peaks at the end of exercise [32], [37] To this end, we have established a DNA bank with 285 participants to facilitate future cohort studies.

## Conclusion

The substitution of glutamic acid at position 531 by lysine in the CPT1B protein decreases the mitochondrial beta-oxidation pathway, which increases the RQnp value during recovery from exercise. This may in turn contribute to either weight gain or a reduced weight-loss following exercise.

## Supporting Information

File S1 **Contains Tables S1–S5.** Table S1: Birthplace of the participants who completed the experimental tests. Table S2: Participants distribution in each comparison groups. Table S3: Mean (SD) values of heart rate (HR) in Tecumseh Step Test. Table S4: Means values and (SD) of blood indicators according with genetic classification. Table S5: Means and (SD) of blood hormones concentrations and hematocrit according with genetic classification.
(DOCX)
