# Supplementary material for: Carnitine Palmitoyltransferase 1B 531K Allele Carriers Sustain a Higher Respiratory Quotient after Aerobic Exercise, but β3-Adrenoceptor 64R Allele Does Not Affect Lipolysis: A Human Model
Source: PLoS One. 2014 Jun 6;9(6):e96791. doi: 10.1371/journal.pone.0096791 (PMC4048163; doi:10.1371/journal.pone.0096791)
Supplement: File S1 — Contains Tables S1–S5. Table S1: Birthplace of the participants who completed the experimental tests. Table S2: Participants distribution in each comparison groups. Table S3: Mean (SD) values of heart rate (HR) in Tecumseh Step Test. Table S4: Means values and (SD) of blood indicators according with genetic classification. Table S5: Means and (SD) of blood hormones concentrations and hematocrit according with genetic classification. (DOCX) [file pone.0096791.s001.docx]

***Supplementary Data.***

| Table S1: Birthplace of the participants who completed the experimental tests. | | | | | | |
| --- | --- | --- | --- | --- | --- | --- |
| Birthplace (State) | Selected participants | Completed test. |  | Renounce |  | %. |
| Colima | 33 | 26 |  | 7 |  | 78.8 |
| Jalisco | 6 | 5 |  | 1 |  | 83.3 |
| Michoacán | 4 | 4 |  | 0 |  | 100 |
| Estado de México. | 1 | 1 |  | 0 |  | 100 |
| DF | 1 | 1 |  | 0 |  | 100 |
| **Total.** | **45** | **37** |  | **8** |  | **82.2** |

| Table S2: Participants distribution in each comparison groups. | | | | | | |
| --- | --- | --- | --- | --- | --- | --- |
| Genetic classification. |  | Comparison groups. |  | Men. | Women. | Total. |
|  |  |  |  |  |  |  |
| ADRB3 alleles |  | R Carriers vs.  R Non-carriers. |  | 10  8 | 10  9 | 20  17 |
|  |  |  |  |  |  |  |
|  |  |  |  |  |  |  |
| CPT1B alleles |  | K Carriers vs.  K Non-carriers |  | 13  5 | 14  5 | 27  10 |
|  |  |  |  |  |  |  |
| Allelic Interaction. |  | K & R Carriers vs.  Only K Carriers vs.  Only R Carriers vs.  K & R Non-carriers. |  | 6  7  4  1 | 6  8  4  1 | 12  15  8  2 |
|  |  |  |  |  |  |  |
| **N** | | | | **18** | **19** | **37** |

| Table S3. Mean (SD) values of heart rate (HR) in Tecumseh Step Test. | | | | | | | |
| --- | --- | --- | --- | --- | --- | --- | --- |
| HR 1 minute post exercise. | G531K CPT1B | | W64R ADRB3 | | ADRB3–CPT1B Interaction. | | |
|  | K (+) | K (-) | R (+) | R (-) | K &R | Only K | Only R |
|  | 85.89 (14.9) | 85.9 (15.0) | 88.4 (15.4) | 82.9 (13.7) | 89.8 (14.8) | 82.7 (14.7) | 86.2 (17.0) |
|  | *p = 0.998* | | *p = 0.266* | | *p = 0.681* | | |
| ANOVA one way. Significance p < 0.05  (+) Allele Carriers, (-) Allele Non Carriers. | | | | | | | |

| Table S4. Means values and (SD) of blood indicators according with genetic classification. | | | | | | | | | | | |
| --- | --- | --- | --- | --- | --- | --- | --- | --- | --- | --- | --- |
| Genetic Classification. | | Rest. | 2’ post exercise | 15’ post exercise. | 30’ post exercise. | Genetic Classification. | | Rest. | 2’ post exercise | 15’ post exercise. | 30’ post exercise. |
| Lactate (mmol/L) | K Carriers  K Non carriers | 1.77 (0.50)  1.76 (0.61) | 2.07 (0.71)  2.06 (0.93) | 1.46 (0.49)  1.58 (0.64) | 1.37 (0.55)  1.45 (0.48) | Glucose (mg/dL) | K Carriers  K Non carriers | 90.2 (10.7)  88.5 (11.3) | 88.8 (10.1)  83.0 (8.1) | 85.0 (9.7)  83.5 (8.8) | 85.4 (8.9)  87.4 (12.0) |
|  |  | *p = 0.973* | *p = 0.982* | *p = 0.532* | *p = 0.689* |  |  | *p = 0.679* | *p = 0.110* | *p = 0.663* | *p = 0.579* |
|  | R Carriers  R Non carriers | 1.64 (0.49)  1.91 (0.55) | 2.10 (0.77)  2.02 (0.77) | 1.49 (0.54)  1.49 (0.54) | 1.37 (0.49)  1.42 (0.58) |  | R Carriers  R Non carriers | 89.4 (11.6)  90.1 (10.0) | 87.3 (10.7)  87.2 (8.9) | 85.1 (10.9)  84.1 (7.5) | 87.2 (11.1)  84.4 (7.7) |
|  |  | *p = 0.119* | *p = 0.766* | *p = 0.992* | *p = 0.741* |  |  | *p = 0.843* | *p = 0.996* | *p = 0.741* | *p = 0.391* |
|  | K-R Carriers  Only K carriers  Only R carriers | 1.63 (0.54)  1.87 (0.46)  1.65 (0.42) | 2.05 (0.64)  2.08 (0.78)  2.18 (0.99) | 1.38 (0.41)  1.51 (0.56)  1.65 (0.68) | 1.33 (0.49)  1.41 (0.61)  1.43 (0.50) |  | K-R Carriers  Only K carriers  Only R carriers | 90.0 (13.1)  90.3 (9.0)  88.5 ( 9.8) | 89.9 (11.6)  87.9 (8.9)  83.3 (8.4) | 86.2 (11.8)  84.13 (7.89  83.5 (9.7) | 86.3 (10.0)  84.7 (8.2)  88.6 (13.3) |
|  |  | *p = 0.383* | *p = 0.834* | *p = 0.697* | *p = 0.942* |  |  | *p = 0.982* | *p = 0.427* | *p = 0.923* | *p = 0.784* |
| Albumin. (micro mol/L) | K Carriers  K Non carriers | 611.8 (69.7)  615.4 (92.0) | 672.1 (82.0)  659.5 (61.9) | 542.0 (38.0)  571.0 (66.8) | 535.2(77.2)  515.3 (79.0) | Glicerol. (micro m/L) | K Carriers  K Non carriers | 73.90 (22.7)  95.57 (45.1) | 224.7 (124.7)  282.2 (113.2) | 141.6 (68.0)  154.5 (43.0) | 91.8 (33.5)  96.2 (26.5) |
|  |  | *p = 0.898* | *p = 0.664* | *p = 0.105* | *p = 0.493* |  |  | *U, p = 0.206* | *U, p = 0.230* | *U, p = 0.286* | *U, p = 0.393* |
|  | R Carriers  R Non carriers | 602.6 (68.3)  624.7 (82.6) | 659.0 (61.1)  680.1 (91.9) | 557.5 (54.2)  540.9 (39.9) | 509.5 (77.5)  553.7 (71.7) |  | R Carriers  R Non carriers | 86.74 (34.3)  71.54 (25.8) | 237.0 (100.0)  244.1 (148.5) | 141.6 (51.5)  149.1 (73.8) | 91.37 (26.3)  94.88 (37.3) |
|  |  | *p = 0.378* | *p = 0.410* | *p = 0.304* | *p = 0.082* |  |  | *p = 0.142* | *p = 0.864* | *p = 0.720* | *p = 0.741* |
|  | K-R Carriers  Only K carriers  Only R carriers | 600.0 (61.4)  621.2 (76.4)  606.5 (82.0) | 655.3 (58.0)  685.4 (96.9)  664.4 (69.2) | 551.6 (37.7)  534.3 (37.6)  566.2 (74.7) | 515.5 (81.1)  550.9 (72.9)  500.4 (76.2) |  | K-R Carriers  Only K carriers  Only R carriers | 74.84 (16.6)  73.14 (27.2)  104.6 (46.4) | 200.5 (85.9)  244.0 (148.9)  291.6 (99.4) | 131.7 (55.4)  149.4 (77.7)  156.5 (44.3) | 86.7 (24.2)  95.9 (39.7)  98.4 (29.5) |
|  |  | *p = 0.788* | *p = 0.723* | *p = 0.278* | *p = 0.347* |  |  | *p = 0.069* | *p = 0.462* | *p = 0.837* | *p = 0.838* |
| ANOVA, one-way, signifficance p < 0.05.  U, Mann-Whitney Test. Signifficance p < 0.05.  K, Kruskal-Wallis Test. Signifficance p < 0.05. | | | | | | | | | | | |

| Table S5. Means and (SD) of blood hormones concentrations and hematocrit according with genetic classification. | | | | | | | | | | | |
| --- | --- | --- | --- | --- | --- | --- | --- | --- | --- | --- | --- |
| Classification. | | Rest. | 2’ post exercise | 15’ post exercise. | 30’ post exercise. | Classification. | | Rest. | 2’ post exercise | 15’ post exercise. | 30’ post exercise. |
| Ephinefrine (pg/mL) | K Carriers  K Non carriers | 78.1 (38.3)  65.4 (20.5) | 80.67 (65.8)  96.0 (40.7) | 52.8 (23.9)  66.0 (29.7) | 54.0 (27.2)  53.1 (18.2) | Norephinefrine (pg/mL) | K Carriers  K Non carriers | 381.8 (124.3)  370.2 (77.6) | 712.4 (365.4)  578.3 (202.6) | 317.3 (167.0)  272.9 (75.5) | 268.6 (106.0)  248.7 (81.6) |
|  |  | *U, p = 0.489* | *U, p = 0.128* | *U, p = 0.166* | *U p = 0.493* |  |  | *p = 0.785* | *p = 0.282* | *p = 0.427* | *p = 0.595* |
|  | R Carriers  R Non carriers | 71.9 (34.9)  77.9 (35.0) | 82.0 (40.9)  88.1 (77.8) | 57.8 (26.5)  54.7 (25.8) | 55.65 (27.3)  51.6 (22.2) |  | R Carriers  R Non carriers | 359.1 (123.4)  401.7 (97.1) | 621.8 (319.3)  740.1 (345.2) | 275.5 (142.6)  340.4 (151.2) | **230.6 (73.9)***  **301.7 (113.1)*** |
|  |  | *p = 0.607* | *p = 0.762* | *p = 0.727* | *p = 0.627* |  |  | *p = 0.257* | *p = 0.287* | *p = 0.188* | ***p = 0.028*** |
|  | K-R Carriers  Only K carriers  Only R carriers | 73.2 (42.8)  82.0 (35.3)  70.0 (20.5) | 68.2 (36.1)  90.7 (82.3)  102.8 (40.9) | 54.6 (29.3)  51.3 (19.5)  62.5 (22.7) | 54.7 (33.5)  53.5 (22.2)  57.1 (16.2) |  | K-R Carriers  Only K carriers  Only R carriers | 351.7 (150.6)  405.9 (97.4)  370.3 (74.1) | 624.6 (386.5)  782.6 (344.5)  617.6 (204.8) | 272.3 (175.6)  353.2 (156.4)  280.1 (81.8) | 214.2 (63.1)  312.2 (114.7)  255.1 (86.1) |
|  |  | *p = 0.566* | *p = 0.606* | *p = 0.448* | *p = 0.797* |  |  | *p = 0.672* | *p = 0.365* | *p = 0.450* | *p = 0.066* |
| Insuline (uU/mL) | K Carriers  K Non carriers | 19.6 (17.4)  26.6 (17.6) | 15.4 (7.4)  14.7 (7.4) | 12.3 (8.9)  17.1 (13.1) | 9.8 (6.5)  13.8 (11.0) | Grow hormone(ng/mL) | K Carriers  K Non carriers | 1.06 (2.0)  2.28 (3.0) | 4.69 (3.2)  5.44 (2.5) | 2.33 (1.8)  2.49 (1.2) | 1.47 (1.2)  1.21 (0.7) |
|  |  | *p = 0.287* | *p = 0.794* | *p = 0.217* | *p = 0.178* |  |  | *U, p = 0.199* | *U, p = 0.374* | *U, p = 0.584* | *U, p = 0.811* |
|  | R Carriers  R Non carriers | 24.5 (19.2)  18.0 (15.1) | 15.7 (6.8)  14.6 (8.0) | 15.0 (9.4)  12.1 (11.2) | 12.3 (9.2)  9.2 (6.2) |  | R Carriers  R Non carriers | 1.73 (3.0)  0.99 (1.4) | 5.4 (3.1)  4.3 (2.8) | 2.8 (1.7)  1.9 (1.5) | 1.6 (1.0)  1.2 (1.1) |
|  |  | *p = 0.262* | *p = 0.654* | *p = 0.397* | *p = 0.245* |  |  | *U, p = 0.647* | *U, p = 0.259* | *U, p = 0.120* | *U, p = 0.259* |
|  | K-R Carriers  Only K carriers  Only R carriers | 25.0 (21.7)  15.4 (12.2)  23.9 (16.0) | 17.5 (7.6)  13.8 (7.0)  13.2 (4.6) | 15.5 (11.1)  9.8 (6.0)  14.1 (6.8) | 11.4 (7.4)  8.5 (5.6)  13.6 (11.8) |  | K-R Carriers  Only K carriers  Only R carriers | 1.26 (2.8)  0.90 (1.3)  2.44 (3.3) | 4.95 (3.6)  4.48 (2.9)  6.10 (2.3) | 2.76 (2.0)  2.00 (1.5)  2.84 (1.1) | 1.71 (1.2)  1.29 (1.1)  1.39 (0.6) |
|  |  | *p = 0.250* | *p = 0.324* | *p = 0.164* | *0.445* |  |  | *K, p = 0.368* | *K, p = 0.380* | *K, p = 0.407* | *K, p = 0.771* |
| Cortisol (ug/dL) | K Carriers  K Non carriers | 9.61 (5.2)  9.03 (3.6) | 10.90 (6.1)  7.81 (5.6) | 9.42 (5.4)  6.77 (4.7) | 8.59 (5.4)  5.94 (3.9) | Hematocrit (%) | K Carriers  K Non carriers | 43.7 (4.0)  43.9 (6.0) | 43.2 (3.6)  43.1 (5.2) | 41.3 (3.7)  42.2 (5.0) | 41.1 (3.5)  41.4 (4.9) |
|  |  | *p = 0.752* | *p = 0.172* | *p = 0.181* | *p = 0.165* |  |  | *p = 0.891* | *p = 0.975* | *p = 0.540* | *p = 0.0.825* |
|  | R Carriers  R Non carriers | 9.35 (4.8)  9.57 (4.9) | 10.32 (6.9)  9.77 (5.1) | 8.94 (5.9)  8.43 (4.7) | 7.68 (5.1)  8.10 (5.2) |  | R Carriers  R Non carriers | 44.0 (5.1)  43.3 (3.8) | 43.2 (4.7)  43.1 (3.2) | 41.7 (4.5)  41.3 (3.7) | 41.4 (4.3)  40.9 (3.4) |
|  |  | *p = 0.892* | *p = 0.787* | *p = 0.777* | *p = 0.805* |  |  | *p = 0.698* | *p = 0.917* | *p = 0.767* | *p = 0.755* |
|  | K-R Carriers  Only K carriers  Only R carriers | 9.19 (5.5)  9.94 (5.1)  9.59 (3.8) | 11.48 (7.5)  10.44 (5.0)  8.58 (6.1) | 9.88 (6.4)  9.06 (4.7)  7.53 (5.0) | 8.85 (5.6)  8.38 (5.4)  5.92 (4.1) |  | K-R Carriers  Only K carriers  Only R carriers | 44.2 (4.4)  43.3 (3.7)  43.8 (6.3) | 43.3 (4.4)  43.0 (3.1)  43.0 (5.5) | 41.6 (4.0)  41.0 (3.6)  41.9 (5.3) | 41.6 (3.8)  40.7 (3.4)  41.0 (5.3) |
|  |  | *p = 0.857* | *p =0.449* | *p = 0.439* | *p = 0.588* |  |  | *p = 0.958* | *p = 0.996* | *p = 0.865* | *p = 0.851* |

ANOVA, one-way, significance: p < 0.05. **U**, Mann-Whitney Test, significance: p < 0.05. **K**, Kruskal-Wallis Test, significance: p < 0.05.
